# Increased erosion of high-elevation land during late Cenozoic: evidence from detrital thermochronology off-shore Greenland

**DOI:** 10.1038/s41598-022-14129-6

**Published:** 2022-06-15

**Authors:** Valerio Olivetti, Silvia Cattò, Massimiliano Zattin

**Affiliations:** Department of Geosciences, University of Padova, Padua, Italy

**Keywords:** Geomorphology, Sedimentology

## Abstract

Mountain regions at high altitudes show deeply incised glacial valleys that coexist with a high-standing low-relief landscape, whose origin is largely debated. Whether the plateaus contributed to sediment production during the late Cenozoic is a currently debated issue in glacial geomorphology and paleoclimatology. In this study, we used detrital apatite fission-track dating of marine sediments to trace provenance and spatial variation in focused erosion over the last 7 million years. The decomposition of age distributions into populations reveals that, moving upwards through the sections, two young populations get younger, while two older populations get progressively older. We interpreted these trends as the effect of glacial erosion on the valley floors and an increased sediment contribution from the high elevations. To test this hypothesis, we compared the measured ages with synthetic age distributions, which represented a change in the elevation of focused erosion. We conclude that the central-eastern Greenland region is the main source of sediments, and in addition to enhanced valley incision, sediments have also been sourced from progressively higher elevations since 7 Ma. The ageing trend provides an unusual case in detrital thermochronology and a strong evidence that intensified Quaternary glaciations amplify the erosional process both in valley bottoms and at high elevations.

## Introduction

The topography of numerous mountain chains located at high latitudes in both hemispheres, such as the Transantarctic Mountains and the North Atlantic continental margins, is characterized by elevated low-relief surfaces deeply incised by fjords. In Greenland and Norway, the origin of such topography is largely debated, and two end-member hypotheses have been proposed^1,2^. According to the first hypothesis, the low-relief landscape was formed at low elevations during a Mesozoic peneplanation phase and uplifted during the Cenozoic; the low-relief surface thus represents a remnant erosional surface where the present erosion is negligible. According to the second hypothesis, the low-relief surface was formed at high elevations in response to a slow, continuous and ongoing process of widespread erosion at high altitudes. These two hypotheses imply different geological and geomorphological processes and different amounts of sediment production over time, especially in the last few million years. The quantification and variation of sediment production in the late Cenozoic provides a relevant and largely debated issue among Earth scientists. Many studies have indicated an increase in relief and sediment yield during the Quaternary, supporting the idea of a global climate-driven late Cenozoic-enhanced erosion^3,4^, while other studies have reported either steady global erosion rates or unresolved changes, highlighting the uncertainties caused by depositional hiatuses, varying measurement intervals and local tectonic activity^5–7^. One possible source of bias (i.e. spatial variations in tectonic activity) along the Greenland margins is of minor importance, given the general consensus on the occurrence of very little tectonic activity during the Quaternary. Therefore, southeast Greenland is a suitable location to test the impact of Quaternary climate changes on erosion, particularly to assess the possible contribution of higher elevations to sediment production.

**Figure 1:**
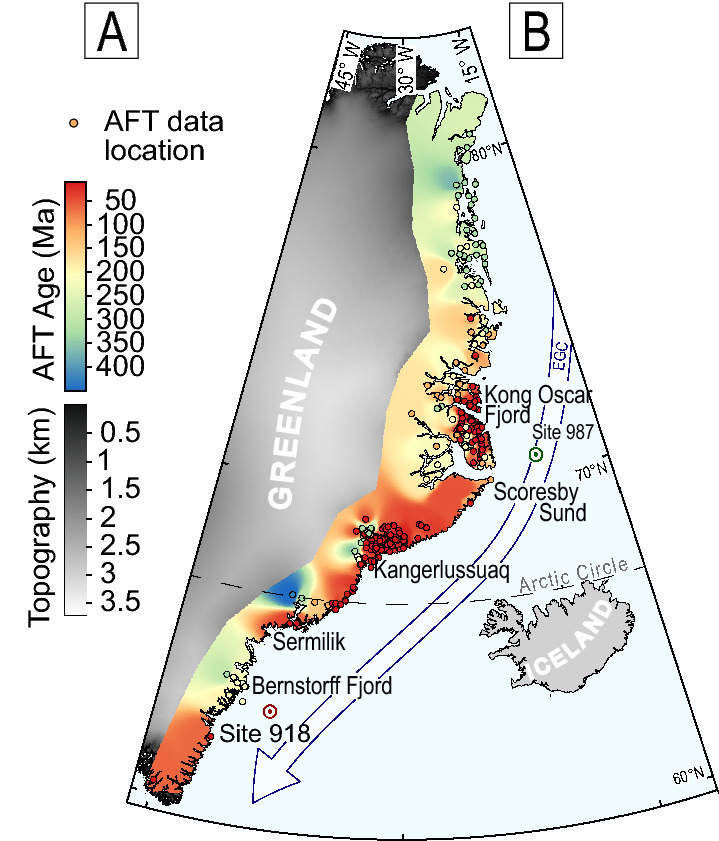
(**A**) DEM of eastern Greenland with interpolation map of in situ fission track ages. Locations of ODP sites used in this study and East Greenland Current are shown. The map was created from GIMP data^14^ using QGIS 3.x software.

To address this issue, we conducted a detrital apatite fission-track (AFT) study on 10 marine sediment samples obtained from two cores (sites 918 and 987)^8,9^ of the Ocean Drilling Project (ODP) (Fig. 1), which cover a depositional time from 7.3 to 0.6 Ma, encompassing the late Cenozoic major shifts in global temperature^10^. At the beginning of this period, an initial glaciation in Greenland had already occurred, and glaciers had reached the coast^11^. Analyses of ice-rafted debris have suggested that the first large-scale glaciation occurred at 3.3 Ma and expanded further by 2.7 Ma^12^, in agreement with the Pliocene–Pleistocene global climate variation. The complete stabilisation of the Greenland Ice Sheet occurred at 0.8 Ma when the magnitude of glaciation and duration of glacial cycles increased^13^.

The effects of glacial valley incision on isostatic uplift were modelled by Medvedev^15^, who inferred a maximum of 1.1 km of surface uplift of the Fjords Mountains (Scoresby Sund, Fig. 1) induced by localised erosion, assuming an onset of glacial incision since 3 Ma and considering limited pre-existing valleys. This amount of erosion is not enough to be recorded in bedrock thermochronological ages (the AFT closure depth is ~ 3 km; the exact amount depends on the geothermal gradient), which instead records the pre-Quaternary exhumation history^16–20^. Even a lower-temperature thermochronometer highlighted limited erosion during the late Cenozoic^21^. Pre-Cenozoic in situ thermochronological ages are particularly common^16^ and have been associated with the erosion of the Caledonian orogen. Cenozoic ages have been found in several discontinuous portions of East Greenland, particularly along a 50-km-wide area parallel to the coast^17,19^. These ages have resulted from the exhumation mainly associated with following rifting, continental breakup and mantle flow^16–19^, with a possible contribution due to late Cenozoic glacial erosion^18^. This study aims to evaluate the contribution of the erosion of high-elevated landscapes to sediment production during the last 7 million years.

## Methods and results

### Detrital fission-track record and lag time

**Figure 2:**
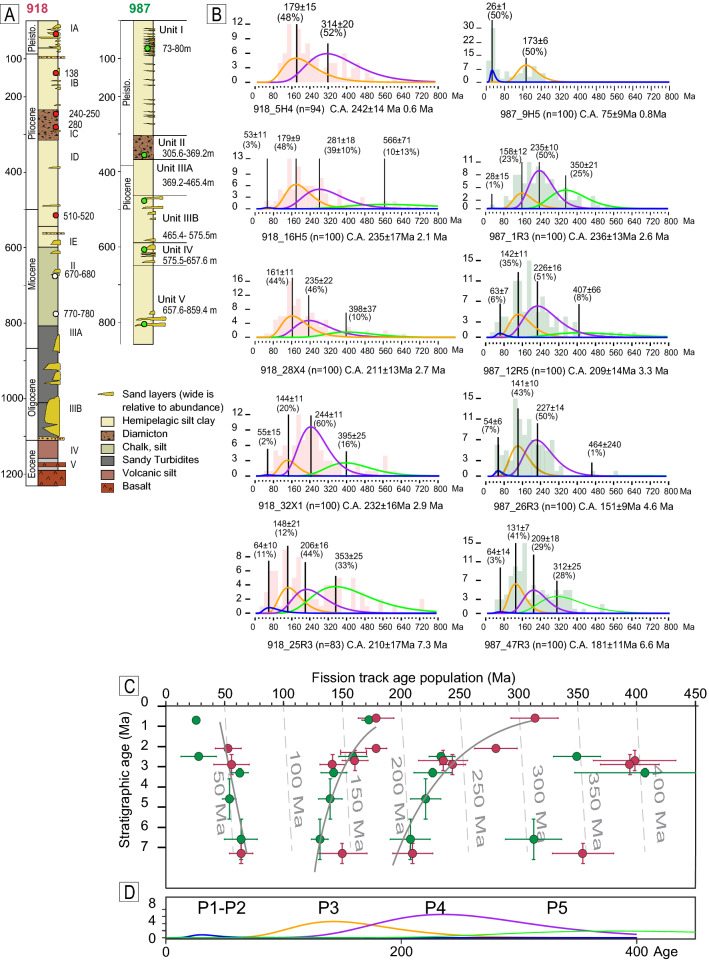
(**A**) Stratigraphic log with locations of sampling spot, in modified from^8,9^. (**B**) Histograms of AFT grain age and decomposition into a population of ages. The number in brackets is the number of dated grains. C.A. is the central age, the age of the undecomposed distribution. (**C**) Lag time plot where the ages of populations of detrital fission-track data are plotted against stratigraphic ages. The samples obtained from site 918 are represented in red, while those obtained from site 987 are represented in green. Curves represent a regression of P2, P3 and P4; see Supplementary Figure S1. Vertical dotted lines represent the same lag time as that corresponding to a constant erosion rate. (**D**) Decomposition into populations of the distribution obtained from the sum of the ages of all samples, using Binomfit^22^.

We analyse, through fission-track thermochronometry, 10 samples of late Miocene to Middle Pleistocene marine sediments originating from two ODP cores—the leg 152 site 918^8^ and the leg 162 site 987^9^ (Fig. 1) drilled in 1994 and 1996, respectively (Figs. 1, 2A).

Fission tracks are damage features in crystals produced by spontaneous nuclear fission of ^238^U and accumulated over time. When the AFT method is used on detrital samples whose temperature does not exceed the thermal sensitivity of the system (60–120 °C) after deposition, we obtain a grain-age distribution that reflects the thermal history of the sediment source and the provenance. The age distribution is usually a mixture assumed to comprise a series of overlapping finite components (i.e. age populations). To decompose the mixture distribution of AFT ages, two types of software are routinely used in thermochronology: one based on binomial peak-fitting (Binomfit^22^) and the other based on a hybrid algorithm that uses both a deterministic and a Markov-chain Monte Carlo approach (Density plotter^23^). The analytical procedure for AFT dating is described in the Methods section, and the analytical data are presented in Table S1.

The mixture distributions of AFT grain age have been decomposed using Binomfit in the automatic mode (i.e., without any a priori setting). The obtained detrital populations (Table 1, Fig. 2B,C) are consistent across the two drill cores, which is surprising considering the distance of > 1000 km between the cores. Samples of similar stratigraphic age from the two drill cores show much similar AFT populations. The two youngest populations (P1: 26–28 Ma, P2: 55–64 Ma) match the in situ AFT ages detected sparsely along the Greenland coasts (e.g., at the Kong Oscar Fjord and Kangerlussuaq) (Fig. 1). In the literature, these young ages are associated with the exhumation caused by the continental margin uplift following the continental breakup and transition over the Iceland hotspot^17,19^.

**Table 1:** Population age.

Sample	Sample depth	Depositional age (Ma)^a^ ± unc	No. of crystals	*P*(χ)^2^	Central Age (Ma) ± 1σ	P1		P2		P3		P4		P5	
						Age ± Unc	(%)	Age ± Unc	(%)	Age ± Unc	(%)	Age ± Unc	(%)	Age ± Unc	(%)
987B_9H5	77.00–77.30	0.7 ± 0.1	100	0	80.9 ± 7.3	26 ± 1	50			173 ± 6	50				
987E_1R3	366.76–367.06	2.5 ± 0.1	100	0	233.7 ± 10	28 ± 15	1			159 ± 12	24	235 ± 11	50	350 ± 20	25
987E_12R5	471.58–471.88	3.3 ± 0.2	100	0	187.4 ± 10			63 ± 7	6	143 ± 11	37	227 ± 16	49	408 ± 60	8
987E_26R3	603.25–603.55	4.6 ± 1	100	0	166.5 ± 8.4			54 ± 6	7	140 ± 10	42	221 ± 13	48	646 ± 200	2
987E_47R3	805.41–805.71	6.6 ± 1	100	0	186.5 ± 9.5			64 ± 14	3	131 ± 7	40	208 ± 17	29	313 ± 24	27
918A_5H4	35.03–35.33	0.6 ± 0.1	94	0	239.3 ± 12					179 ± 15	48	314 ± 20	52		
918A_16H5	139.37–139.67	2.1 ± 0.1	100	0	230.6 ± 13			53 ± 11	3	179 ± 9	48	281 ± 18	39	566 ± 67	10
918A_28X4	249.21–249.51	2.7 ± 0.5	100	0	204.8 ± 9.9					161 ± 11	52	236 ± 21	38	399 ± 35	10
918A_32X1	279.75–280.05	2.9 ± 0.5	100	0	227.3 ± 12			56 ± 15	2	142 ± 11	21	244 ± 11	62	395 ± 25	15
918D_25R3	514.55–514.85	7.3 ± 0.5	83	0	209.0 ± 14			64 ± 10	11	150 ± 21	15	210 ± 17	43	355 ± 26	31

^a^Depositional age and relative uncertainties are estimated from the age model refined by Bierman et al.^13^.

The pre-Cenozoic age populations (P3 and P4) are in agreement with the widespread in situ AFT ages in southeastern Greenland. In both cores, P3 (144–180 Ma) is the more recurring and abundant population (Fig. 2B). In contrast, P4 is not well defined, as it spans over a large age interval (206–314 Ma); furthermore, it is not always present. P3 and P4 do not correspond to any specific tectonic or thermal events known in the literature^16^ because the Greenland bedrock was characterized by slow exhumation during the late Paleozoic and Mesozoic. Thus, their origin in the detrital record cannot be directly linked to a specific source area of provenance.

The comparison of AFT data with depositional ages provides the classical lag-time plot (Fig. 2C). Depositional ages are derived from the refined age model of Bierman^13^ for site 918 and 987 drill cores.

Although the age of the populations of each sample changes over time, the populations remain clearly discernible. Therefore, the trend of each population through time can be reconstructed.

To support the identified populations shown in Fig. 2C, we merge all grain ages of each sample into a single distribution and decompose this distribution into populations (Fig. 2D). The four obtained populations are similar to the populations of each sample, suggesting that the gathering of Fig. 2C is statistically solid. Moreover, in supplementary information, we show that when other population gatherings are taken into account, the statistic worsens (Fig. S1). The trend of P1 population is not taken into account in the two samples only. The second youngest population, P2, gets younger moving up through the section, representing the classical trend produced by a constant (or nearly increased) erosion of a crustal block (Fig. 2C). They are probably evidence of progressive deepening of the valley due to glacial erosion. The two older populations (P3 and P4) show an unexpected trend of ageing, which seems to increase at ~ 3 Ma (Fig. 2C). Such a trend is rarely found in the literature^24^. The ageing trend is also observable in the trend of central ages of each sample (Fig. 2B), which tend to get older moving towards the younger stratigraphic age.

## Discussion

### Provenance of pleistocene sediments

**Figure 3:**
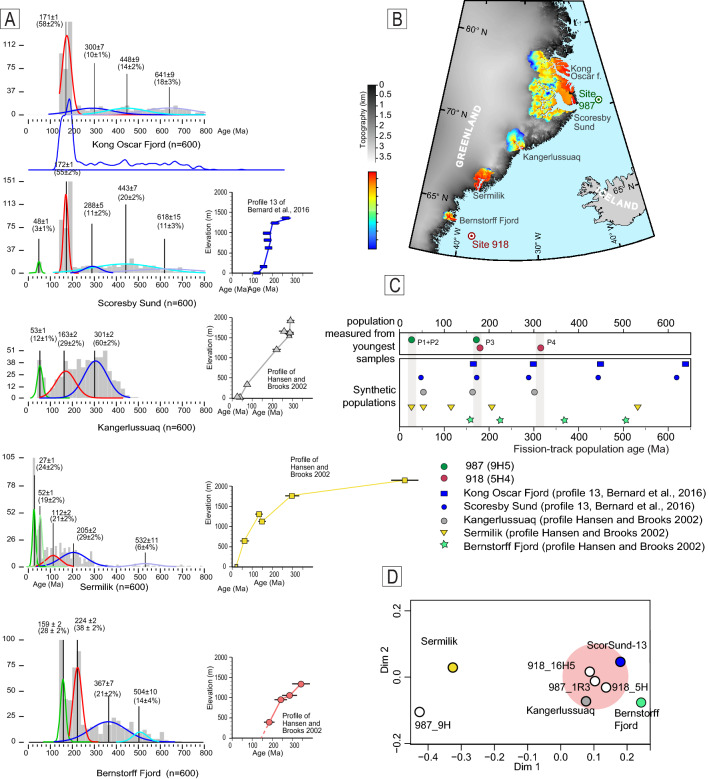
(**A**) Synthetic age distribution and populations for the five drainage areas. Histograms of the synthetic ages are shown in grey in the background; the age populations after decomposition are shown in colour. Vertical black lines and numbers represent the position of the populations, age and proportion with uncertainties (from Density Plotter^22^). Age-elevation profiles used to calculate synthetic age distribution are shown nearby. Profiles are vertically aligned to indicate the differences. For Scoresby Sund and Kong Oscar Fjord, the same elevation-age profile is used. (**B**) Map of southeastern Greenland, which shows drainage areas used to calculate the synthetic age distributions. The distribution of the synthetic age is shown in colour. The map was created using ArcMap 10.5 software. (**C**) Comparison of the age of the measured population of the two youngest samples (middle Pleistocene) with that of the synthetic populations. Populations form Kong Oscar Fjord, Scoresby Sund, and Kangerlussuaq match well with the measured P3 and P4 populations, which are marked in grey. (**D**) MDS plot, where the distance between the samples reflects the similarity of the distribution. The entire distribution is considered, and not just the age of a single population. The plot shows that three of the youngest measured samples are similar to the synthetic samples of Scoresby Sund and Kangerlussuaq.

Sediments of the Greenland shelf are sourced from a few large fjords and transported by the Greenland Current^25^. Our P1 population (26–28 Ma) is particularly abundant in the youngest sample of site 987 and matches well with the widespread AFT ages occurring at the mouth of Kong Oscar Fjord^18,20^ (Figs. 1, 3B). For older populations, the comparison of detrital age with in situ ages does not allow a direct identification of the source area because of the lack of unambiguous signature in bedrock age distribution. We, therefore, compare our data with the synthetic populations calculated for the five largest fjords of southeastern Greenland (Fig. 3B). Synthetic age distribution has been generated by convolving catchment hypsometry with published AFT ages detected along the vertical profiles^16,18^ so that an AFT age is assigned to each pixel of a digital elevation model (DEM from GIMP^14^), following the approach proposed in Reference^26^. A spatially uniform erosion has been assumed; therefore, each pixel of the DEM contributes equally to the age distribution. We use 600 random points to produce a mixture distribution, which we decompose into age populations (Fig. 3A) using the Density Plotter software^23^. We compare synthetic with measured distributions in two ways: by comparing the ages of single components (populations) of the mixture distribution with the measured components (Fig. 3C) and by using a multidimensional scaling (MDS) analysis (Fig. 3D).

The shape of each synthetic distribution is a result of basin hypsometry and the AFT age-elevation profile. For Bernstorff, Sermilik and Kangerlussuaq, only one age-elevation profile is available in the literature for each area, while for Scoresby and Kong Oscar Fjord, many profiles are available^18^. We select the profile with the maximum difference in elevation (n13^18^).

Synthetic age populations from Scoresby Sund, Kong Oscar Fjord and Kangerlussuaq fit well with the measured detrital age population (Fig. 3C) found in the youngest samples. We then note that the synthetic distribution of Scoresby Sund and Kong Oscar Fjord yields a population age that matches well with that measured population age, although the distribution is highly dispersed and the components are discernable only after software decomposition.

MDS analysis allows the comparison of the entire distribution and not only the age of single populations (Fig. 3D). Three of the four youngest measured distributions are plotted close to the synthetic distribution obtained by Scoresby Sund and Kangerlussuaq, indicating the similarity between these distributions. Sample 987_9H5 is plotted far due to its distribution characterized by a relatively high quantity of young AFT ages (Fig. 3D).

As a further output, P3 and P4 populations can be generated by the erosion of the same catchment when large differences in AFT ages occur with elevation. This pattern is especially evident when a break in slope in the age–elevation relationship is present^27^. In other words, the finding of P3 and P4 in the detrital record does not necessarily imply the presence of two different sources.

Different results are obtained from the southernmost catchments, where synthetic populations match less well with the measured samples (Fig. 3C), making it unlikely that sediments of site 918 are sourced by proximal sources, such as Sermilik or Bernstorff Fjord. This finding agrees with the Holocene sediment source studies^25^, which consider Scoresby Sund and subordinated Kong Oscar Fjord and Kangerlussuaq as the largest sources of sediments that drift hundreds of kilometres with the southward-directed East Greenland Current.

### Population age trend through time

**Figure 4:**
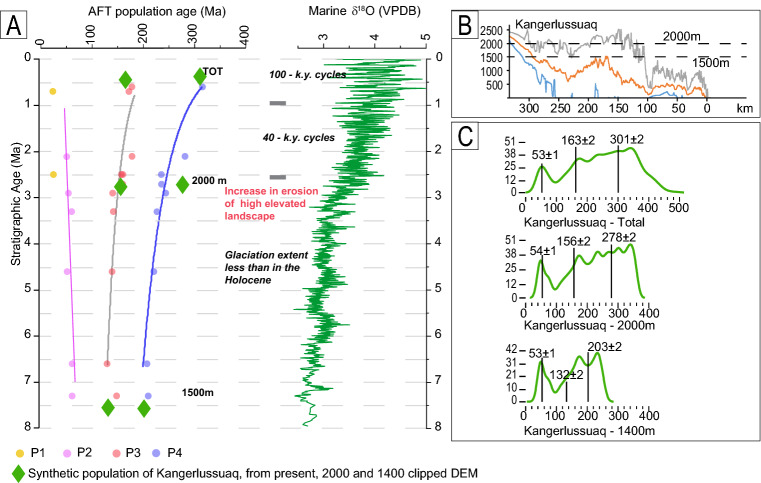
(**A**) AFT population age lag time for ODP sites 987 and 918 compared to a global δ^18^O curve^8^. Samples show P1 and P2 populations, which get younger moving upwards, whereas P3 and P4 show an increasing age towards the younger stratigraphic age. P3 and P4 trends over time mimic the δ^18^O curve, suggesting a climatic control in the detrital AFT signal. Measured population ages are compared with synthetic ages obtained from the Kangerlussuaq drainage area, which is reduced in elevation to test the increasing contribution of the sediment obtained from a high elevated landscape. (**B**) Swath profile of Kangerlussuaq: the profile starts from the coast line towards inland; lines represent minimum, mean and maximum elevations; dotted lines indicate the elevation at which the DEMs are clipped. (**C**) Synthetic probability distribution and age of populations obtained from drainage areas reduced to 2000 and 1500 m in elevation.

The progressive ageing of P3 and P4 peaks is the main outcome of our analyses. This trend of AFT detrital ages contrasts with the expected trend for an eroding and exhuming source, where the continuous unroofing by glaciers is expected to result in progressively younger detrital AFT ages. The trends of P3 and P4 populations mimic the global marine δ^18^O record^10^ (Fig. 4A), suggesting a climatically controlled process of sediment production; a climatic origin is even more convincing when we observe that ageing in P3 and P4 populations increases at 2.5–3 Ma, thus corresponding to the first growth of a full ice sheet in Greenland^13^.

A simple possible mechanism that can explain the increase in the age population moving upwards in the section is a constant increase in the sediments with old AFT ages derived from higher altitudes. Therefore, the ageing trend is evidence of a constantly higher erosion efficiency at high altitudes. This hypothesis is based on the following conditions: (1) in situ AFT ages get older from coast to higher elevations, following the typical pattern for passive margins; (2) P3 and P4 are produced through the erosion of the same source area; and (3) no large changes in apatite fertility occur in the same source region. At first approximation, these conditions seem verified for the study area. First, the published bedrock AFT age-elevation profiles show a clear increase in age with the elevation and moving inland from the coast^16,18,28^. The low slope of age–elevation correlation, resulting from the slow exhumation, makes AFT ages well-constrained for variation in elevation, providing the best condition to study a sediment source in relation to altitude^29^. The comparison between synthetic and measured age populations for Pleistocene samples, as previously shown, testifies that a wide range of ages can be generated by erosion of the same source area, especially when a large difference in age with elevation exists^27^. The quantification of possible apatite fertility variations in the source area is much more difficult. In the first approximation, fertility depends on lithology. However, AFT age and lithology are not related because a large part of Paleozoic sediments record the same exhumation history as that recorded by crystalline rocks. Thus, an increase in old AFT age grains cannot be associated with focused erosion of a specific lithology.

The enhanced efficiency of erosion processes at high altitudes from high-latitude regions has been postulated in pioneering hypotheses over a century ago (e.g. by Penck, see Brozović^30^); recently, it has been verified by numerical modelling^1,31,32^ and supported by geological and thermochronological evidence^29^. Our findings highlight that the erosion of the high elevated landscape and interfjord uplands occurs at the same time as the classical channelized incision of deep valley and fjords. These data are totally consistent with Steer^33^, which suggests a great contribution of the high-elevation landscape in sediment production since approximately 3 Ma to explain the volume of marine sediments in Norwegian offshore. Our ageing trend of P3 and P4 populations does not imply a unique contribution from a high elevated landscape but only a relative increase in grains with old AFT ages. The trend of P1 and P2 shows that glacial deepening of the valley floor is constant and contributes to providing younger AFT age through time. The younging trend of P1 and P2 populations is evidence of active glacial incision. The difference in the magnitude of age variation between the slow younging of P1–P2 and the fast ageing of P3 and P4 is noteworthy. This fast ageing is not directly associated with an increased erosion rate, but only with an increased quantity of grains with older AFT ages. The slow younging of P1 and P2 reflects the slow erosion rate associated with the glacial process that is considered quite slow during the late Cenozoic^21^. We note a possible decrease in erosion rate due to the cold-based glacier effect and larger cover of ice sheet during late Cenozoic cannot produce ageing in the AFT age trend. As reported in the literature^24,34^, a decrease in erosion rate produces a vertical lag time line and never an inverted line, as shown in Fig. 4A.

Highly efficient erosion mechanisms at high altitudes concern periglacial processes; geomorphic processes for increased periglacial erosion around and above the equilibrium line altitude have been invoked by many authors^30,35,36^. These processes, such as glacial cirque headward and frost cracking, are particularly efficient during interglacial periods. An increase in the contribution of old AFT grains in our populations can be a result of glacial and interglacial cycles, because each sample covers a time window corresponding to many glacial cycles, due to the length of the sampling spot. This interpretation is supported by the evidence that between 2.5 and 0.8 Ma, the ice did not constantly cover the source areas^13^.

To test how the detrital age distribution changes in response to a variation in elevation of the source sediments, we produce a series of synthetic AFT age distributions. Synthetic distribution has been created from DEM, whereby higher elevations are removed (following the approach of Ehlers^29^). Synthetic detrital ages have been produced from the catchments of Kangerlussuaq (Fig. 4) and Kong Oscar Fjord (Fig. S2) following three steps of clipping: (1) a present-day topography (the same as that used for provenance analysis), (2) a reduced-elevation DEM clipped at 2000 m and (3) a reduced-elevation DEM clipped at 1500 m asl (Fig. 4B). The distribution of AFT ages from clipped DEMs is expected to simulate the detrital age pattern resulting from focused erosion during three moments: an old scenario where erosion was focused below 1500 asl, an intermediate step (2000 m clipped DEM) and the present day. The amount of erosion since 7 Ma (the bottom of the stratigraphic section) has been arbitrarily chosen with the aim to reproduce, only qualitatively, the general trend of ageing. Although we use an erosion amount that is not based on the measured data, it is consistent with the erosion rate derived from the onshore–offshore mass balance study of approximately 100 m/Myr^33^, although considered too high by other authors^37^.

The trend of the synthetic population for the Kangerlussuaq fits well the measured data and, in the case of clipped catchment at 1500 m, the synthetic populations reproduce well the measured population from the lowermost samples (Fig. 4C). The synthetic trend derived from Kong Oscar Fjord (Fig. S2) shows a similar trend of age variation, although the fit with the measured data is not so strong. The comparison between the synthetic and measured ages, although qualitative, confirms that an enhanced contribution of sediments with old AFT ages makes the AFT populations older moving towards the stratigraphycally younger samples. In conclusion, the modelling of detrital data shown in this study provides a reliable explanation for the ageing trend of the AFT population, supporting the idea of a progressively enhanced erosion of a high-standing landscape, as proposed by the glacial buzzsaw and isostasy-climate-erosion (ICE) hypothesis^1^.

## Method

### Thermochronological analysis

Apatite grains were separated from approximately 0.3 kg of the drill core sample using standard heavy liquids and a magnetic technique. Mounts were grounded, polished and etched with 5% HNO_3_ at 20 °C for 50 s to reveal the spontaneous tracks. They were coupled with the external detector mode on a low-U muscovite sheet. The samples were then irradiated with thermal neutrons in the Radiation Center of the Oregon State University. After irradiation, the low-U muscovite detectors were etched in 40% HF at 20 °C for 45 min to reveal the induced fission tracks. AFT ages were measured and calculated using the external detector and zeta calibration methods^38^ with a zeta value (referred to Fish Canyon Tuff and Durango apatite standards^39^) of ξ = 355.02 ± 4.36 (Silvia Cattò analyst) for dosimeter CN5.

### Synthetic thermochronological ages

A DEM of catchments representing sediment sources has been extracted from GIMP DEM^14^, and 600 points have been randomly extracted using GIS integrated tools in ArcMap and QGIS software. An R script converts the elevations of DEM-extracted points into thermochronological ages according to the age-elevation distribution of AFT ages supplied by the user and chosen from published papers.

A series of elevation-age points are inset and used to create segments of the profile. The scrip reads the elevation of the 600 points, places each elevation in the correct segment of the age-elevation profile and determines the corresponding thermochronological age using the line from the two-point formula solved for x, where the two points are the endpoints of the segment. The synthetic thermochronological age and elevation are indicated by x and y, respectively. Synthetic thermochronological ages are decomposed using the Density Plotter software^23^ because of its capability to treat considerable data.

## Supplementary Information

Supplementary Information 1.

Supplementary Information 2.

## Data Availability

Raw data of the single sample track counting are available in a single file in the extra Materials.
