# Supplementary material for: Increased erosion of high-elevation land during late Cenozoic: evidence from detrital thermochronology off-shore Greenland
Source: Sci Rep. 2022 Jun 15;12:9932. doi: 10.1038/s41598-022-14129-6 (PMC9200846; doi:10.1038/s41598-022-14129-6)
Supplement: Supplementary file 1 — Supplementary Information 1. [file 41598_2022_14129_MOESM1_ESM.docx]

**Increased erosion of high-elevation land during late Cenozoic: evidence from detrital thermochronology off-shore Greenland**

Valerio Olivetti, Silvia Cattò, Massimiliano Zattin

**Supplementary Information**

*Summary of Contents*

Table S1: apatite fission track analytical data.

Figure S1: Regression analysis.

Figure S2: Synthetic data from Scoresby Sund to test the effect of change in elevation of sediment source.


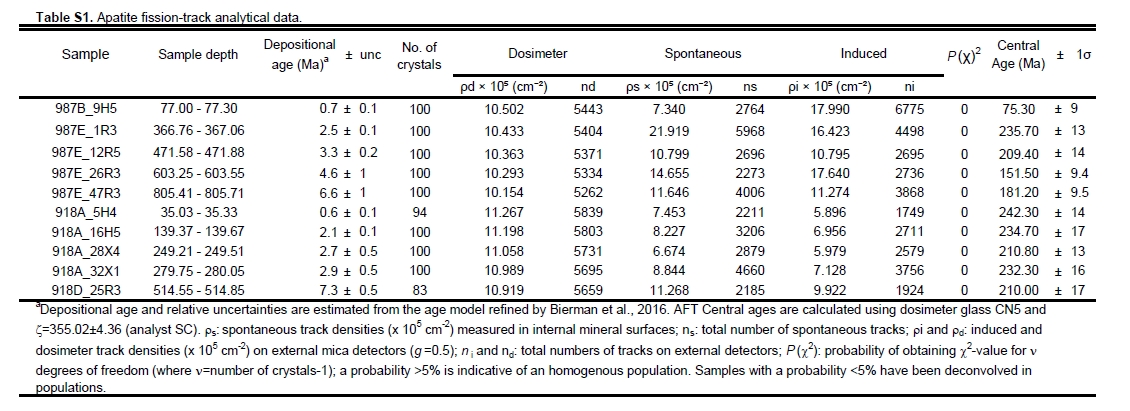


Table S1.


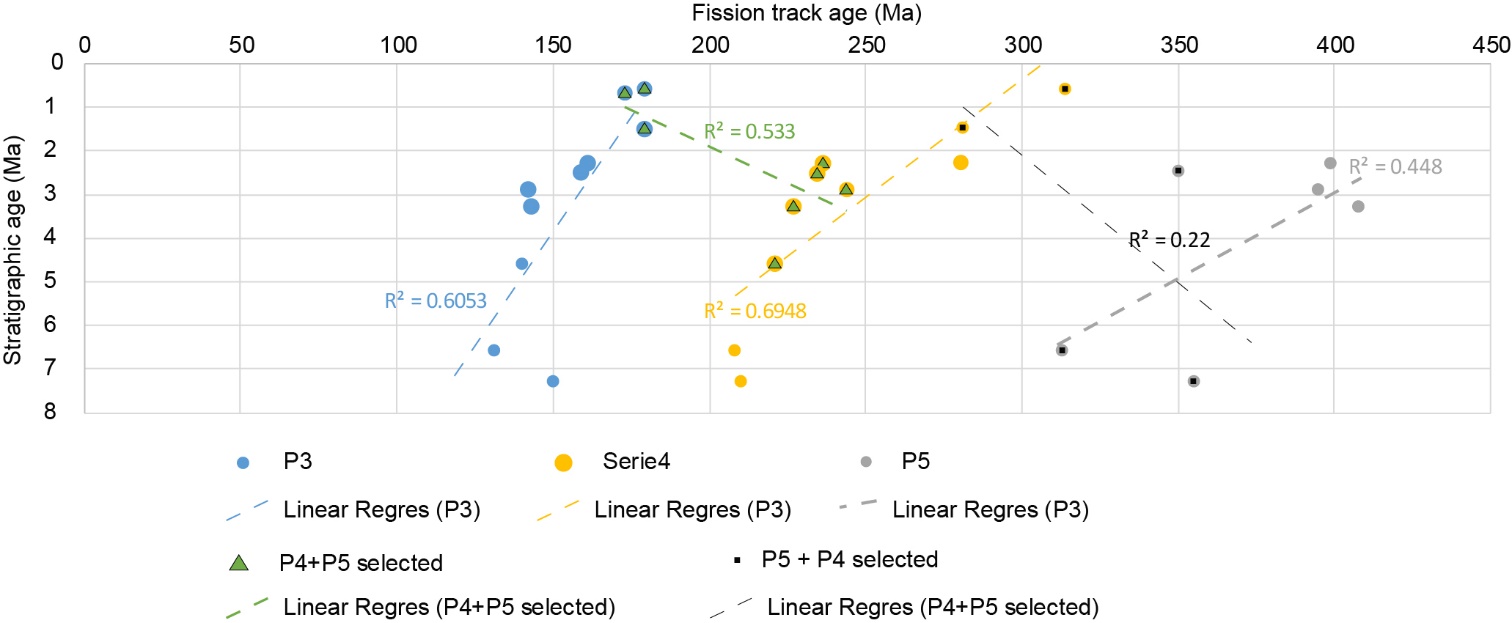


Figure S1. Lag time plot.

Lag time plot for P3, P4 and P5 population. The diagram shows the AFT ages of three populations plotted against the stratigraphic age. We show many regression lines calculated for groups of ages testing different composed of different populations to show the most probable group

The blue, yellow and grey dots represent the division of population as shown in the main test for P3, P4 and P5 respectively. Triangle and square represent an alternative division of age to compose trends. We note that alternative division show data more dispersed and statically less probable. We used the linear regression to compare the simplest models.


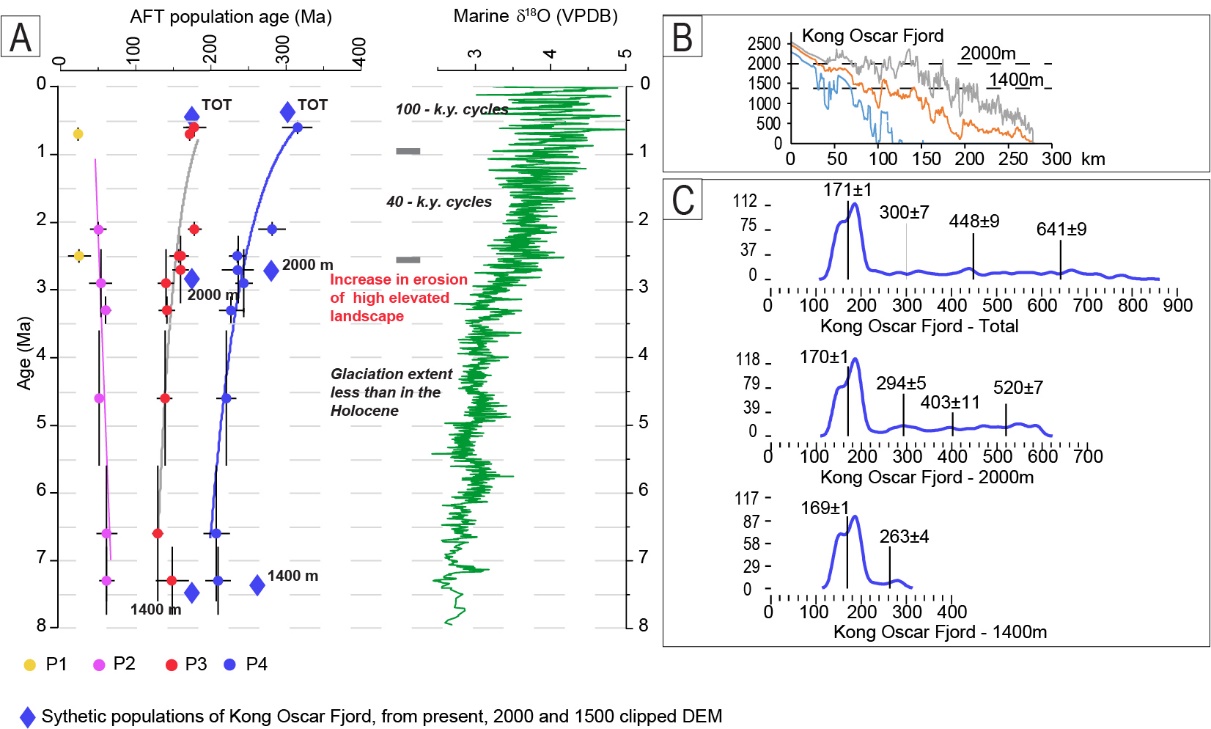


Figure S2.

Similar of Fig. 4 of the main text and relative to Scoresby Sund data.

AFT population age lag time for ODP sites 987 and 918 compared with a global δ^18^O curve^8^. Samples show P1 and P2 population that get younger moving upward whereas P3 and P4 show an increasing age toward the younger stratigraphic age. P3 and P4 trend through time mimics the δ^18^O curve suggesting a climatic control in detrital AFT signal. Measured population ages are compared with synthetic ages obtained from Scoresby Sund drainage area that has been reduced in elevation to test the increasing contribution of sediment from high elevated landscape. (b) Swath profile of the Scoresby Sund: the profile starts from the coast line toward inland, lines represent minimum, mean and maximum elevations, dotted lines are the elevation at which the DEM were clipped. (c) Synthetic probability distribution and age of populations obtained from drainage areas reduced to 2000 and 1500 m in elevation.
